# USP13 Facilitates the Proliferation of Hepatocellular Carcinoma Cells by Reducing K48/63‐Linked Polyubiquitination and Degradation of PRPF6

**DOI:** 10.1111/jcmm.70551

**Published:** 2025-04-10

**Authors:** Yanyu Jiang, Qing Luo, Xuanchao Zhang, Weichao Yang, Renhao Wang, Qinghe Hu, Zhiyi Liu, Bin Zhang

**Affiliations:** ^1^ General Surgery Department The Affiliated Hospital of Xuzhou Medical University Xuzhou Jiangsu China; ^2^ Institute of Digestive Diseases Xuzhou Medical University Xuzhou Jiangsu China; ^3^ Anesthesiology Department The Affiliated Hospital of Xuzhou Medical University Xuzhou Jiangsu China

**Keywords:** deubiquitination, HCC, proliferation, PRPF6, USP13

## Abstract

Ubiquitin‐specific peptidase 13 (USP13) is a well‐characterised deubiquitinating enzyme that plays a critical role in the pathogenesis and progression of various human malignancies. However, the precise mechanisms by which USP13 influences hepatocellular carcinoma (HCC) cell proliferation remain to be fully elucidated. In this study, we confirmed that USP13 expression was upregulated in HCC and correlated with poor prognosis. Further investigation revealed that the knockout of USP13 inhibited HCC cell proliferation, whereas overexpression of USP13 had the opposite effect. Mechanistically, pre‐mRNA processing factor 6 (PRPF6) was identified as a potential substrate of USP13 through mass spectrometry analysis. USP13 stabilised the PRPF6 protein by reducing its K48/63‐linked polyubiquitination levels and degradation. Ultimately, we demonstrated that the USP13‐PRPF6 axis promoted HCC cell proliferation was closely associated with the activation of the AKT‐mTOR signalling pathway.

## 1 Introduction

Primary liver cancer, predominantly hepatocellular carcinoma (HCC), is one of the most common digestive system malignancies. In China, it ranks second in cancer mortality, with a 5‐year survival rate of about 12% [1, 2]. Systemic therapy is still the primary treatment for advanced hepatocellular carcinoma. Despite advances in targeted therapies and immunotherapies, patient outcomes have not significantly improved [3, 4]. It is crucial to investigate the molecular mechanisms of hepatocellular carcinoma progression and identify more effective therapeutic targets to improve clinical outcomes.

Ubiquitination is a ubiquitous form of post‐translational modification that involves the sequential action of three enzymes: E1 ubiquitin‐activating enzyme, E2 ubiquitin‐conjugating enzyme and E3 ubiquitin ligase. These enzymes mediate the covalent attachment of ubiquitin molecules to substrate proteins. Additionally, deubiquitinating enzymes (DUBs) specifically cleave ubiquitin chains, thereby maintaining the dynamic equilibrium of the ubiquitin system and ensuring cellular protein homeostasis and normal cellular functions [5, 6]. Ubiquitin modification plays a pivotal role in the progression of HCC. Specifically, the E3 ubiquitin ligase FBXO7 facilitates the ubiquitination of PRMT1, thereby inhibiting serine synthesis and tumour growth [7]. Additionally, Ubiquitin Specific Protease 1 (USP1) stabilises CDK5, which regulates mitochondrial fission and metabolic reprogramming, thus promoting HCC progression [8]. BRCC36 is involved in the deubiquitination of HMGCR, and its inhibition can suppress liver cancer cell proliferation [9]. Further investigation into the mechanisms of ubiquitination in HCC may provide a theoretical foundation for identifying effective therapeutic targets.

To date, over 100 deubiquitinating enzymes have been identified and classified into seven families: USP, UCH, OTU, JAMM, ZUFSP, MJD and MINDY, with the USP family comprising the largest number of members [10]. Ubiquitin‐specific peptidase 13 (USP13) plays a pivotal role in regulating cell cycle progression, DNA damage response, antiviral responses and other biological processes [11, 12, 13]. USP13 has been implicated in the development and progression of various malignancies. Specifically, it is highly expressed in lung squamous cell carcinoma, where it promotes tumorigenesis by deubiquitinating c‐Myc [14]. Additionally, USP13 stabilises the autophagy‐related protein ATG5, thereby modulating drug resistance in gastrointestinal stromal tumours [15]. In ovarian cancer, USP13 upregulates ATP citrate lyase (ACLY) via deubiquitination, enhancing fatty acid synthesis and accelerating tumour growth [16]. In liver disease, USP13 delays the progression of non‐alcoholic steatohepatitis by inhibiting the IRHOM2‐associated pathway [17]. Whereas in HCC, hypoxia‐induced expression of USP13 enhances TLR4 deubiquitination and activates the TLR4/MyD88/NF‐κB signalling pathway, thereby accelerating tumour progression [18]. But until now, the precise mechanisms underlying the role of USP13 in HCC remain to be fully elucidated and warrant further investigation.

In this study, we systematically investigated the impact of USP13 on HCC cell proliferation and elucidated the underlying molecular mechanisms. Our findings demonstrate that USP13 stabilises PRPF6 protein levels by attenuating its K48/63‐linked polyubiquitination. Furthermore, the USP13‐PRPF6 axis facilitates HCC cell proliferation via modulation of the mTOR signalling pathway.

## 2 Materials and Methods

### 2.1 Tissues

HCC tissue samples and normal liver tissue samples were collected from the Affiliated Hospital of Xuzhou Medical University (Xuzhou, China). HCC specimens came from patients diagnosed with HCC, while normal liver tissues were from individuals undergoing partial hepatectomy due to liver injury. All samples were stored at −80°C immediately after excision. Clinical human tissues used in this study were approved by the Ethics Review Committee of the Affiliated Hospital of Xuzhou Medical University (approval no.: XYFY2019‐KL129‐01). Informed consent was obtained from all patients before treatment. The study was conducted in accordance with the Declaration of Helsinki. The patients signed an internal regulatory document stating that the remaining samples could be used for academic studies without other informed consent.

### 2.2 Cell Culture

The 293 T cells and human HCC cell lines (Huh7 and MHCC97H) were obtained from the Stem Cell Bank of the Chinese Academy of Sciences (Shanghai, China). The cells were cultured in a humidified incubator at 37°C with 5% CO_2_, using Dulbecco's Modified Eagle Medium (DMEM) supplemented with 10% foetal bovine serum (FBS; Gibco).

### 2.3 Stable Cell Line Construction

The 293 T cells were co‐transfected with the target plasmid and helper plasmids (psPAX2 and pMD2.G) using the High Gene transfection reagent (Abclonal, China). Lentiviral particles were harvested 72 h post‐transfection and subsequently used to infect Huh7 and MHCC97H cells. Forty‐eight hours post‐infection, cells were subjected to selection with 2.5 μg/mL puromycin (Beyotime, Shanghai, China).

USP13 sg1 LentiCRISPRv2GFP: GGGAACCATCACTCCTGACG.

USP13 sg2 LentiCRISPRv2GFP: GTTCTTGTAGACCCTGTCGC.

USP13 sg3 LentiCRISPRv2GFP: GCGACCTGCGAGAAAACCTC.

USP13 sg4 LentiCRISPRv2GFP: GGGAACCATCACTCCTGACG.

USP13 sg5 LentiCRISPRv2GFP: GTTCTTGTAGACCCTGTCGC.

PRPF6 sh pLVX‐shRNA2‐PURO: GCGTACTTCGAGAAGAACCAT.

### 2.4 Western Blot

Equal amounts of protein were analysed by SDS‐PAGE and transferred to a 0.45 μm PVDF membrane (Millipore, Billerica, MA). The membranes were blocked with 3% bovine serum albumin (BSA) and incubated with the primary antibody overnight at 4°C. After washed by 1 × TBST three times, the membranes were incubated with the secondary antibody for 2 h at room temperature. Protein bands were visualised using ECL substrate (Thermofisher, UAS) and detected with a chemiluminescence system (Tanon, Shanghai, China). Band density was quantified using ImageJ (NIH, Bethesda, Maryland, USA), and relative protein levels were normalised to internal controls.

### 2.5 Cell Counting Kit‐8 (CCK‐8) Assay

The cells were seeded into 96‐well plates at a density of 5000 cells per well and cultured in an incubator maintained at 37°C with 5% CO_2_. At the same time each day, 10 μL of CCK‐8 reagent was added to each well, followed by an additional incubation period of 4 h at 37°C. The absorbance was then measured at 450 nm using a microplate reader (Tecan).

### 2.6 Colony Formation Assay

A single‐cell suspension from exponentially growing cells was prepared using conventional methods and counted. Then, 500 cells in 5 mL of medium were seeded into each 6‐cm dish. Then the cells were incubated at 37°C with 5% CO_2_ for 2–3 weeks until visible colonies formed. The medium was discarded, and dishes were rinsed twice with PBS. Cells were fixed with 4% paraformaldehyde for 20 min and washed once with PBS. Staining was done with 0.1% crystal violet for 20 min, followed by two more PBS washes. After air‐drying, colony images were captured.

### 2.7 Co‐Immunoprecipitation (Co‐IP) Assay

Cells were lysed using lysis buffer. Proteins were divided into three groups: Input, IP and IgG. For the Input group, 5× loading buffer was added, heated at 100°C for 10 min, cooled and stored at −20°C. For the IP group, the primary antibody was added to 1 mg protein; for the IgG group, an equivalent amount of isotype‐matched IgG antibody was used. Both groups were incubated with pre‐equilibrated protein A/G magnetic beads (MCE) on a rotating shaker overnight at 4°C, followed by a 4‐h incubation at 4°C. The beads were washed three times with chilled 1× TBS, then 2× loading buffer was added to all samples, which were heated at 100°C for 10 min. Finally, samples were analysed by western blot.

### 2.8 Ubiquitination Assay

The corresponding plasmid was transfected into 293 T or HCC cells. Following a 24‐h incubation period, MG132 (10 μM) was added, and the cells were incubated for an additional 6 h. Thereafter, the cells were harvested and lysed using lysis buffer. The whole cell lysate was subsequently incubated with the corresponding antibody at 4°C overnight, followed by incubation with protein A/G magnetic beads at 4°C for 4 h. All subsequent steps adhered to standard immunoprecipitation protocols.

### 2.9 Statistical Analysis

All experiments were conducted in triplicate or more, and all images were quantified using Image J software. Data analysis was performed using GraphPad Prism version 9.0. The differences between the two groups were assessed using an unpaired *t*‐test. A *p*‐value of less than 0.05 was considered to indicate statistical significance.

## 3 Results

### 3.1 USP13 Is Up‐Regulated in HCC and Associated With Poor Prognosis

**FIGURE 1:**
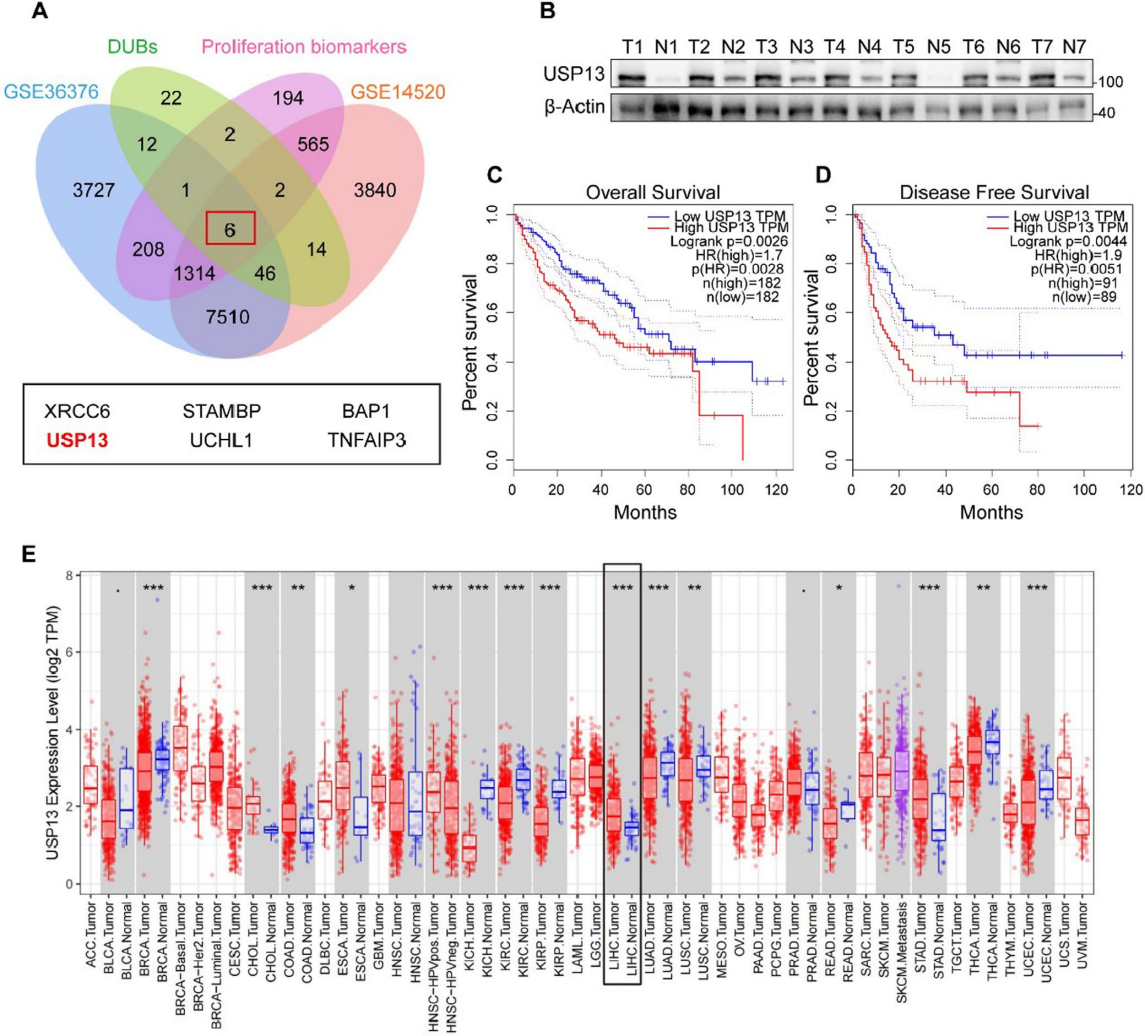
USP13 is up‐regulated in HCC and associated with poor prognosis. (A) The Venn diagram of the GSE36376 and GSE14520 datasets, established proliferation‐related marker proteins, and DUBs. (B) Total proteins isolated from non‐tumour and HCC tissues were analysed by western blot for assessment of USP13. (C, D) High levels of USP13 were associated with poor prognosis in HCC patients. (E) USP13 showed abnormal expression in various human cancers. *p < 0.05, **p < 0.01, ***p < 0.001.

The uncontrolled proliferation of HCC cells is a hallmark of their malignant potential. To identify DUBs potentially associated with this proliferative phenotype, we integrated data from the GSE36376 and GSE14520 datasets, and then cross‐referenced them with established proliferation‐related marker proteins. This comprehensive analysis identified USP13 as a candidate enzyme that may play a critical role in HCC cell proliferation (Figure 1A). To verify the expression of USP13 in HCC, we systematically selected a random sample of seven human HCC tissue specimens and validated that the USP13 protein exhibited significant overexpression in these samples through quantitative analysis (Figure 1B). Next, we found that USP13 was associated with the poor prognosis of HCC patients (Figures 1C,D). In addition, USP13 showed abnormal expression in various human cancers, with significantly elevated levels in HCC (Figure 1E). This suggests USP13 may play an important role in HCC development.

### 3.2 Knockout of USP13 Inhibits the Proliferation of HCC Cells

**FIGURE 2:**
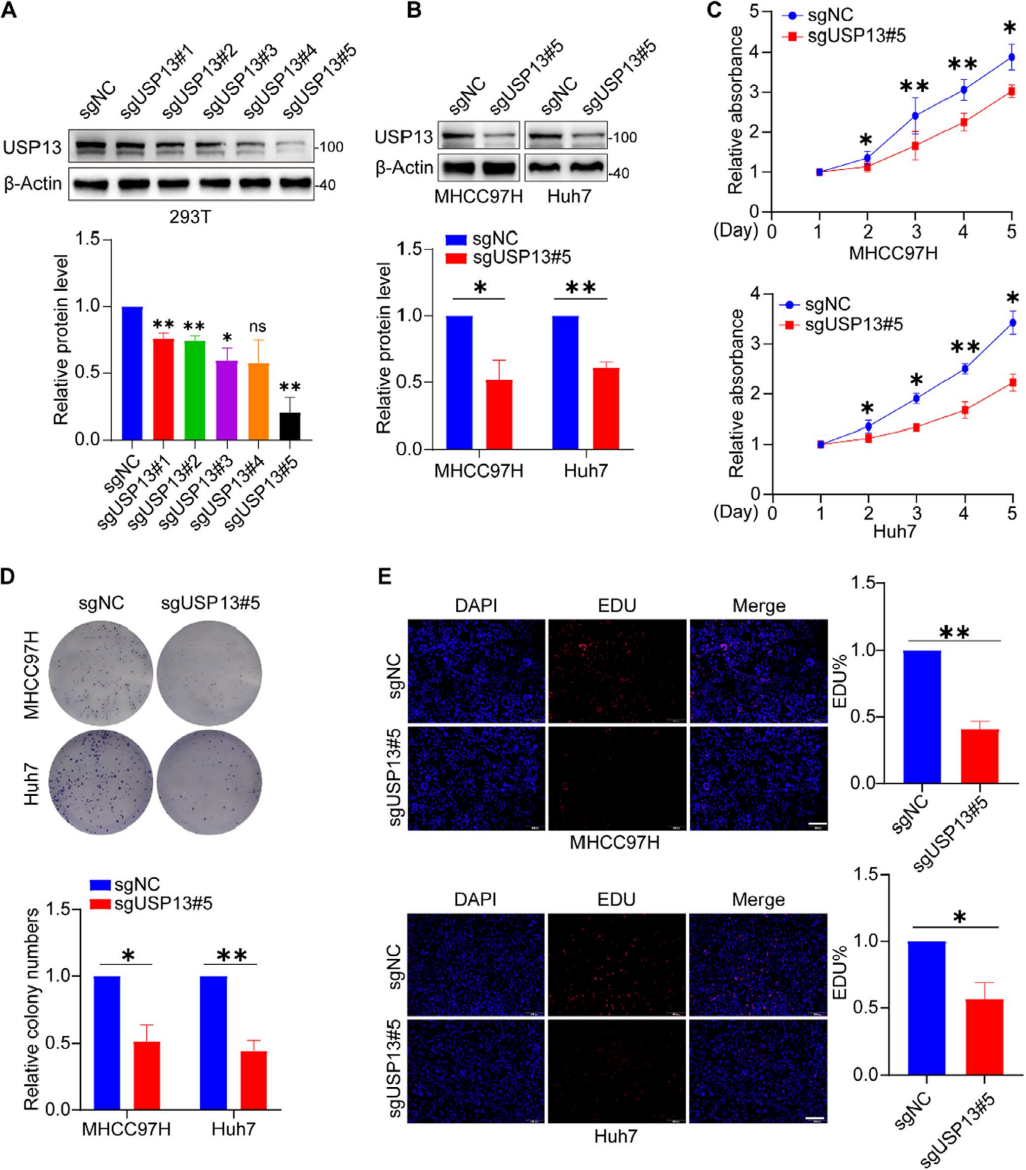
Knockout of USP13 inhibits the proliferation of HCC cells. (A) Representative blots and quantification of sgUSP13 efficiency in 293 T cells. (B) Representative blots and quantification of sgUSP13 efficiency in HCC cells. (C) CCK‐8 assay results. (D) Image and quantification of the colony formation assay with USP13 knocked out HCC cells. (E) Image and quantification of the EdU incorporation assay performed in HCC cells upon knocking out USP13, scale bar: 200 μm. *p < 0.05, **p < 0.01.

To elucidate the relationship between USP13 and HCC cells proliferation, we designed five sgRNA primers targeting USP13 and determined that sgRNA#5 achieved the highest knockout efficiency (Figure 2A). Based on these results, we generated a lentiviral cell line in HCC cells and validated the down‐regulation of USP13 using western blot (Figure 2B). Subsequently, we assessed the growth characteristics of HCC cells following USP13 knockout. The CCK‐8 assay showed that knockout of USP13 obviously reduced cells ability (Figure 2C). Colony formation assays revealed a marked reduction in the colony‐forming capacity of USP13 knockout cells (Figure 2D). The EdU assay revealed a marked decrease in the percentage of EdU‐positive cells subsequent to USP13 knockout (Figure 2E). These results suggest that knockout of USP13 inhibits the proliferation of HCC cells.

### 3.3 Overexpression of USP13 Facilitates the Proliferation of HCC Cells

**FIGURE 3:**
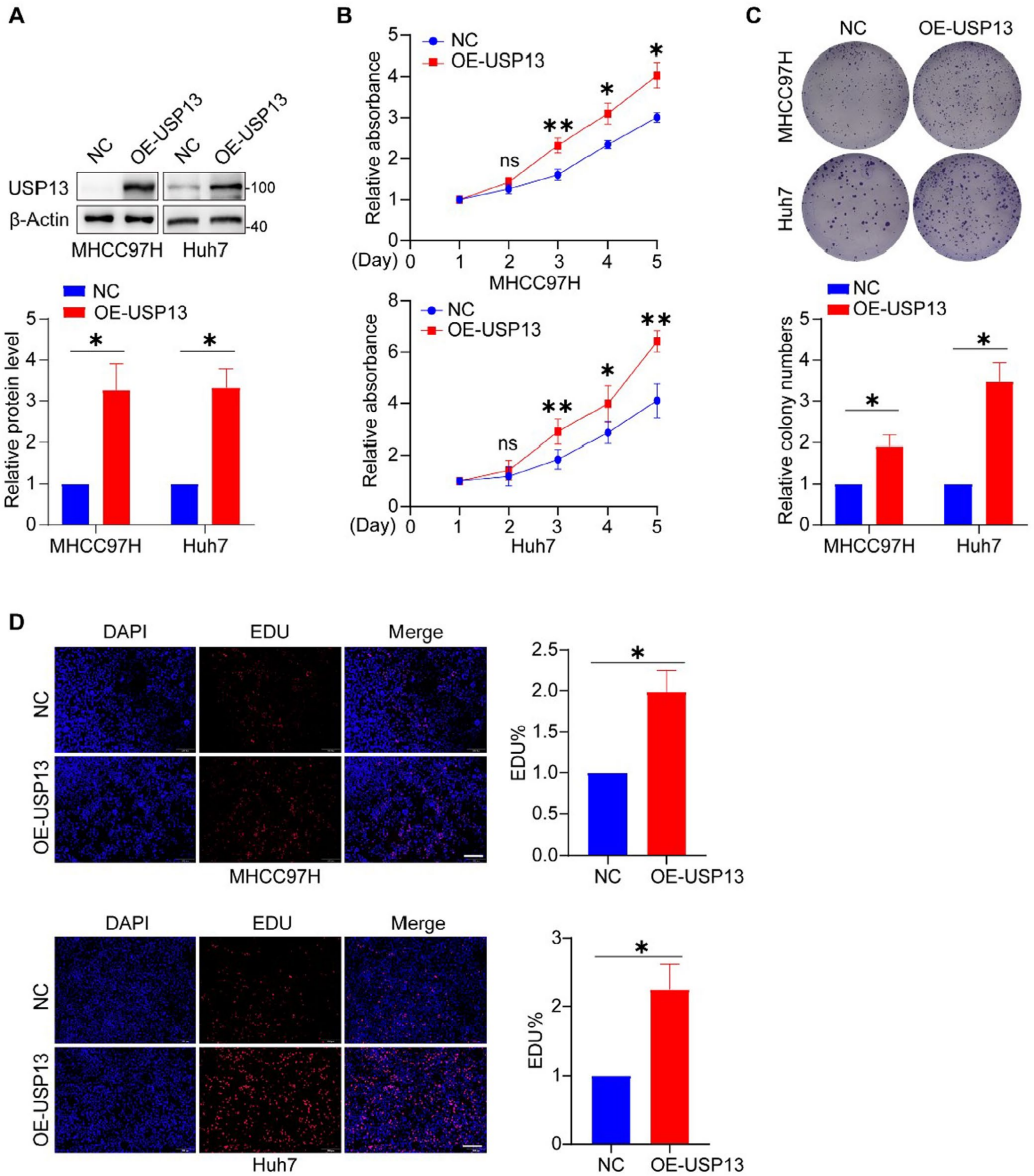
Overexpression of USP13 facilitates the proliferation of HCC cells. (A) Representative blots and quantification of the USP13 overexpression efficiency in HCC cells. (B) CCK‐8 assay results. (C) Image and quantification of the colony formation assay with USP13 overexpressed HCC cells. (D) Image and quantification of the EdU incorporation assay performed in HCC cells upon overexpression of USP13, scale bar: 200 μm. *p < 0.05, **p < 0.01.

To further elucidate the impact of USP13 on the proliferation of HCC cells, we conducted experiments to overexpress USP13 in HCC cell lines and validated the overexpression efficiency using western blot (Figure 3A). Thereafter, we systematically analysed the growth characteristics of HCC cells following USP13 overexpression through a comprehensive set of established experimental methods. In summary, following the overexpression of USP13, the proliferative activity of HCC cells was significantly enhanced, their colony‐forming ability was markedly improved, and the proportion of EDU‐positive cells was substantially increased (Figures 3B–D). These results suggest that overexpression of USP13 promotes the proliferation of HCC cells.

### 3.4 USP13 Stabilises PRPF6 Protein by Decreasing Its K48/63‐Linked Polyubiquitination Levels

**FIGURE 4:**
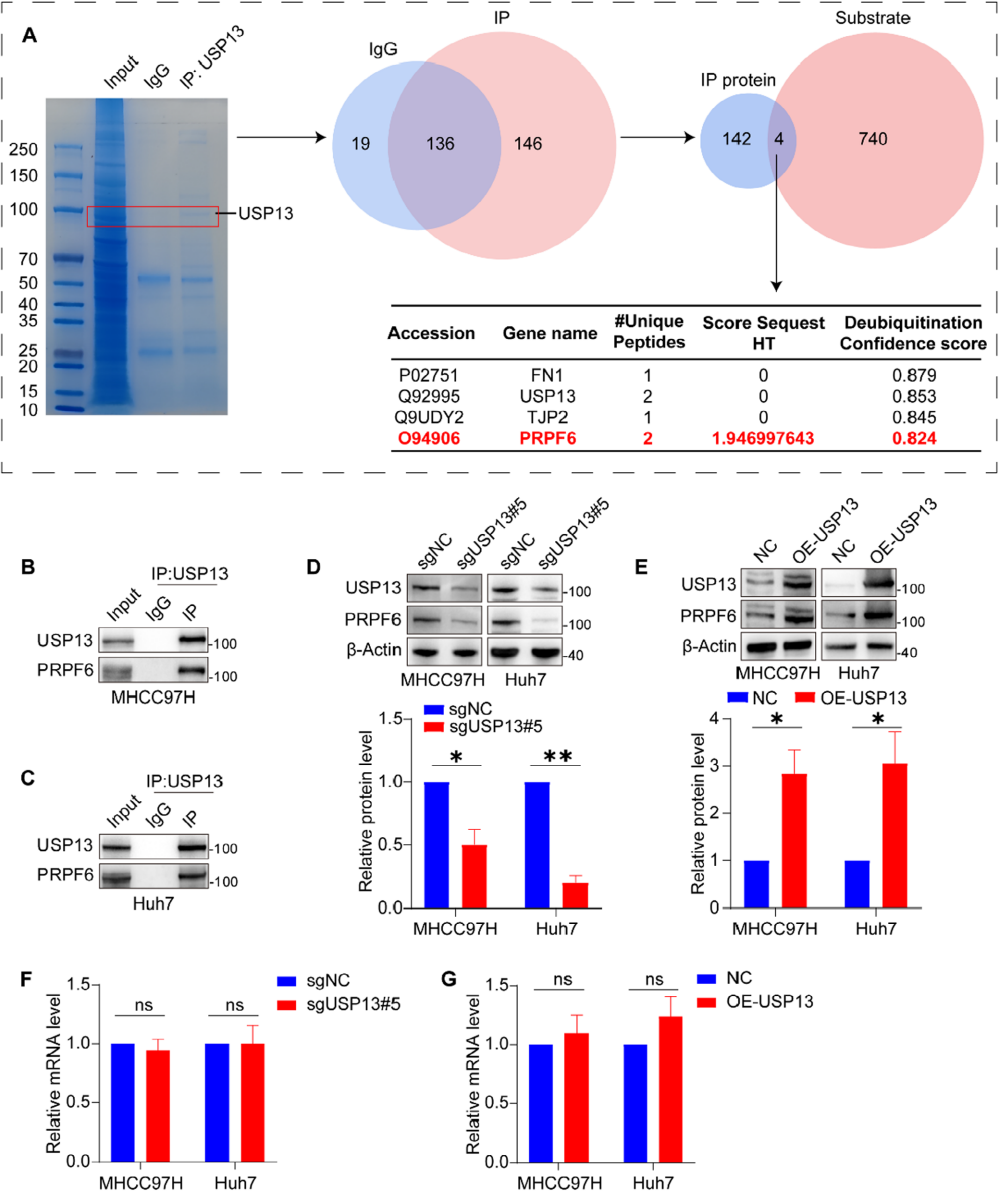
USP13 interacts with PRPF6 and increases its protein level. (A) Schematic representation of PRPF6 as a potential downstream deubiquitylation substrate of USP13. (B, C) Co‐immunoprecipitation assay showed that USP13 interacted with PRPF6 in HCC cells. (D, E) Representative blots and quantification to show the protein level of PRPF6 in USP13 knocked out or overexpressed HCC cells. (F, G) Quantification of PRPF6 mRNA level in USP13 knocked out or overexpressed HCC cells. *p < 0.05, **p < 0.01.

These results above indicate that elevated USP13 expression in HCC promotes cellular proliferation. To elucidate the underlying molecular mechanisms, we utilised mass spectrometry to identify 146 proteins that interact with USP13. We then cross‐referenced these interactors with predicted deubiquitination substrates of USP13 from the Ubibrowser database, leading to the identification of four potential deubiquitination substrates. Based on peptide counts and deubiquitination scores, PRPF6 was selected as the focus of our investigation to determine its role in USP13‐mediated regulation of HCC cell proliferation and to explore the associated mechanisms (Figure 4A). The co‐IP experiment was initially employed to validate the interaction between USP13 and PRPF6. The results demonstrated that USP13 and PRPF6 were components of the same protein complex (Figures 4B,C). Furthermore, we observed that the knockout of USP13 led to a reduction in PRPF6 protein expression, whereas the overexpression of USP13 had an opposite effect (Figures 4D,E). Neither downregulation nor overexpression of USP13 significantly affected PRPF6 mRNA levels (Figures 4F,G). This result further substantiates the hypothesis that PRPF6 may function as a potential deubiquitination substrate for USP13.

**FIGURE 5:**
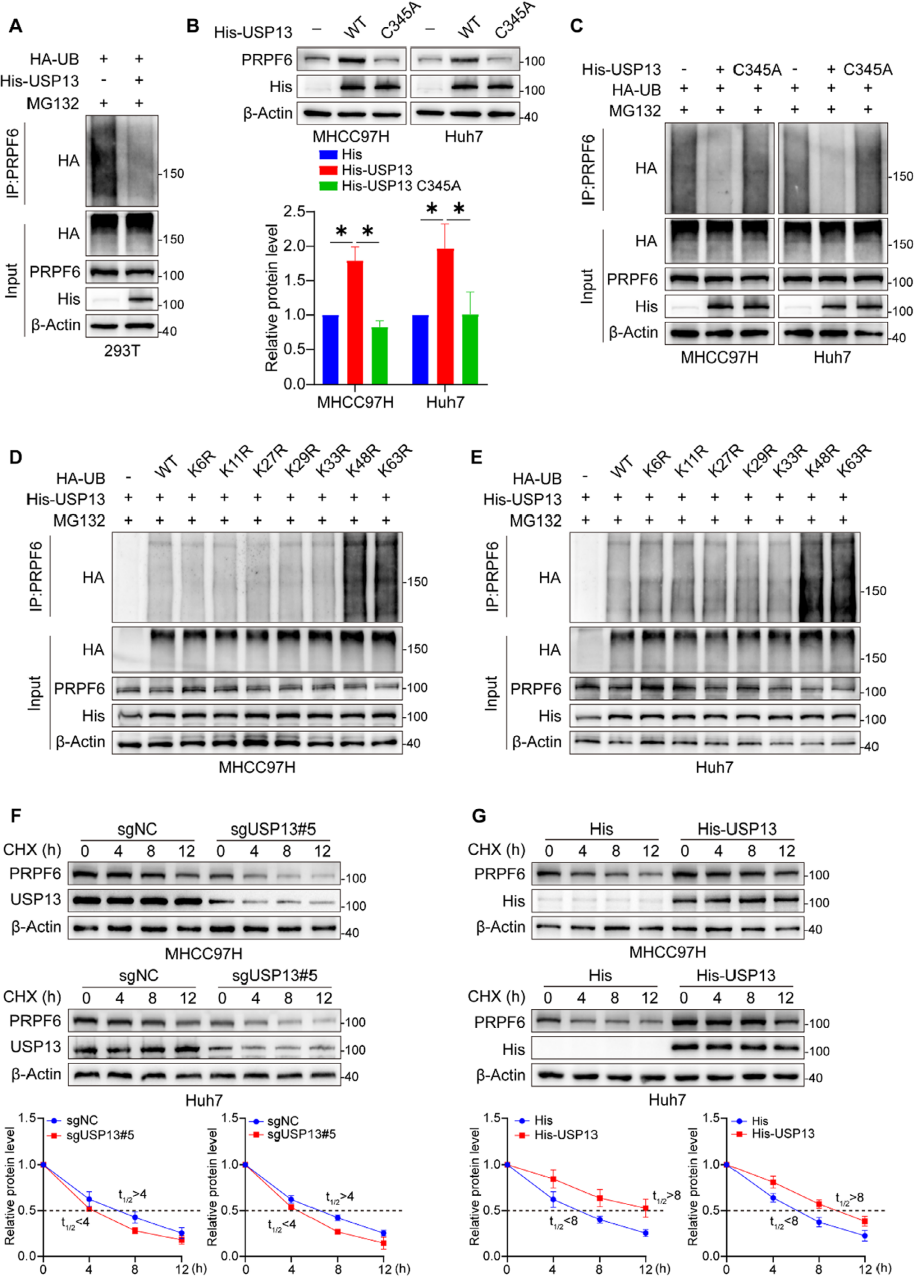
USP13 stabilises PRPF6 protein by decreasing its K48/63‐linked polyubiquitination levels. (A) Representative blots of ubiquitinated PRPF6 in cells overexpressing USP13. (B) Representative blots and quantification of PRPF6 expression in HCC cells with a USP13‐C345A mutation. (C) Representative blots of ubiquitinated PRPF6 in HCC cells with a USP13‐C345A mutation. (D, E) Mutation of Lys48/63 of ubiquitin significantly loses the deubiquitination of PRPF6. (F) Representative blots and quantification showed knockout of USP13 decreased the stability of PRPF6. (G) Representative blots and quantification showed overexpression of USP13 increased the stability of PRPF6. *p < 0.05.

To analyse the molecular mechanism by which USP13 regulates PRPF6, we conducted a detailed investigation into the ubiquitination status of PRPF6 following USP13 overexpression. Our results revealed that USP13 overexpression significantly attenuated the polyubiquitination levels of PRPF6 (Figure 5A). Furthermore, we substituted cysteine at position 345 of USP13 with alanine, thereby generating a mutant that is devoid of deubiquitinating enzyme activity [19]. Encouragingly, the USP13‐C345A mutant exhibited a loss of its ability to modulate PRPF6 protein levels and failed to reduce the polyubiquitination levels of PRPF6 (Figures 5B,C). Based on the linkage mode of ubiquitin molecules, these can be classified into K6, K11, K27, K29, K33, K48 and K63 types. These distinct linkages enable the formation of either homotypic ubiquitin chains or heterotypic branched chains, thereby contributing to the diversity and complexity of ubiquitin modifications [20, 21, 22]. We subsequently determined that USP13 significantly reduced the polyubiquitination of PRPF6, with a specific focus on the K48 and K63 chain linkages (Figures 5D,E). Ultimately, we conducted a comprehensive investigation into the influence of USP13 on PRPF6 protein stability and found that USP13 knockout significantly decreased the half‐life of PRPF6 protein degradation (Figure 5F). In contrast, USP13 overexpression markedly increased PRPF6 protein stability (Figure 5G). These results indicate that USP13 stabilises PRPF6 protein by decreasing its K48/63‐linked polyubiquitination levels.

### 3.5 USP13‐PRPF6 Axis Promotes HCC Cell Proliferation by Regulating AKT–mTOR Signalling

**FIGURE 6:**
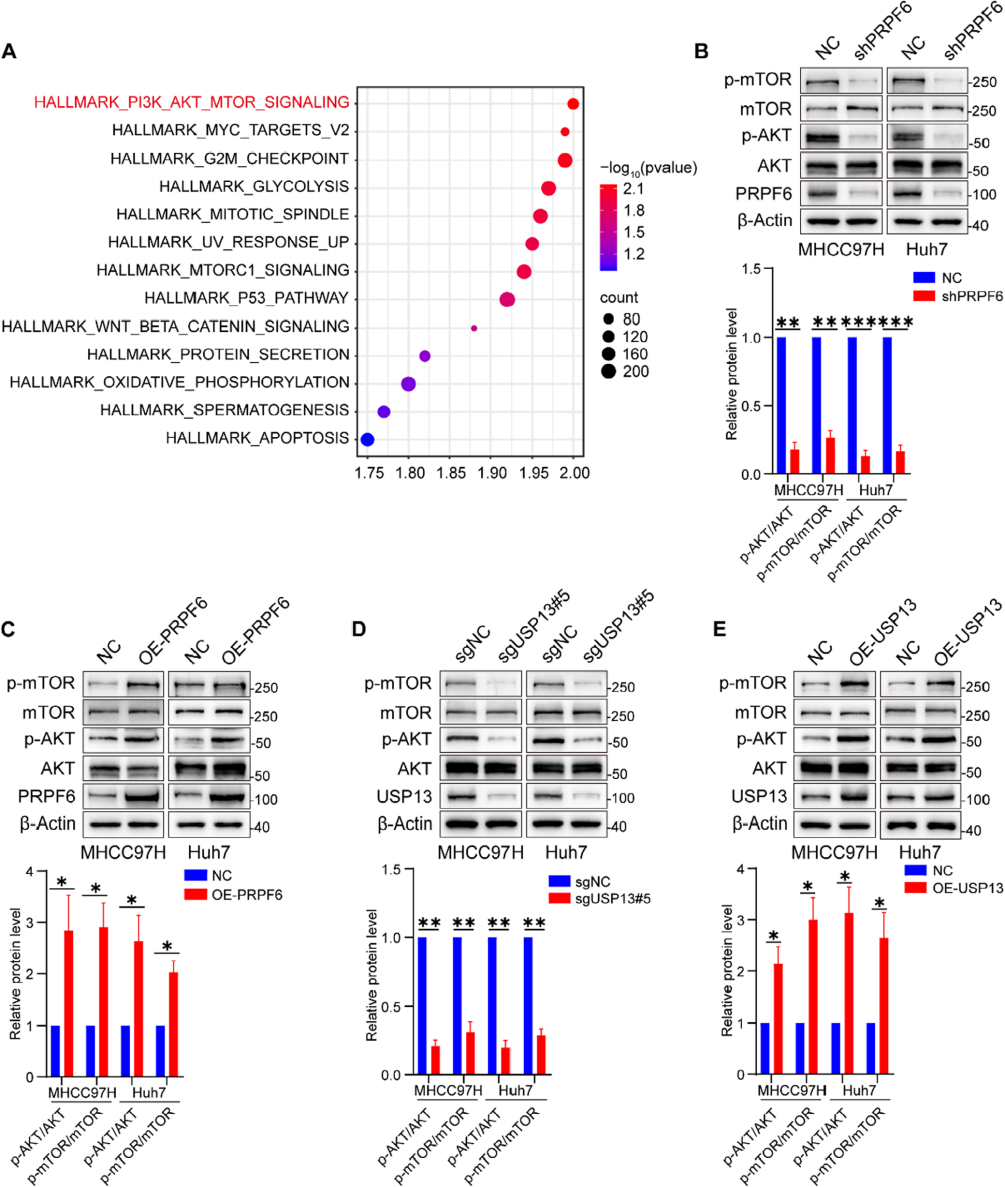
USP13‐PRPF6 axis activates AKT‐mTOR signalling. (A) The image for enrichment pathways. (B, C) Representative blots and quantification to show the protein level of the AKT‐mTOR pathway in PRPF6 knocked down or overexpressed HCC cells. (D, E) Representative blots and quantification to show the protein level of the AKT‐mTOR pathway in USP13 knocked out or overexpressed HCC cells. *p < 0.05, **p < 0.01.

To further explore the signalling pathways underlying the effect of the USP13‐PRPF6 axis on HCC cell proliferation, bioinformatic analysis was performed. Gene set enrichment analysis (GSEA) revealed that PRPF6 was closely related to the AKT‐mTOR pathway (Figure 6A). Subsequently, our findings demonstrated that the overexpression of PRPF6 activated the AKT‐mTOR signalling pathway, whereas the silencing of PRPF6 resulted in an inhibitory effect on this pathway (Figures 6B,C). Importantly, USP13 modulated the AKT‐mTOR signalling pathway in a manner analogous to PRPF6 (Figures 6D,E).

**FIGURE 7:**
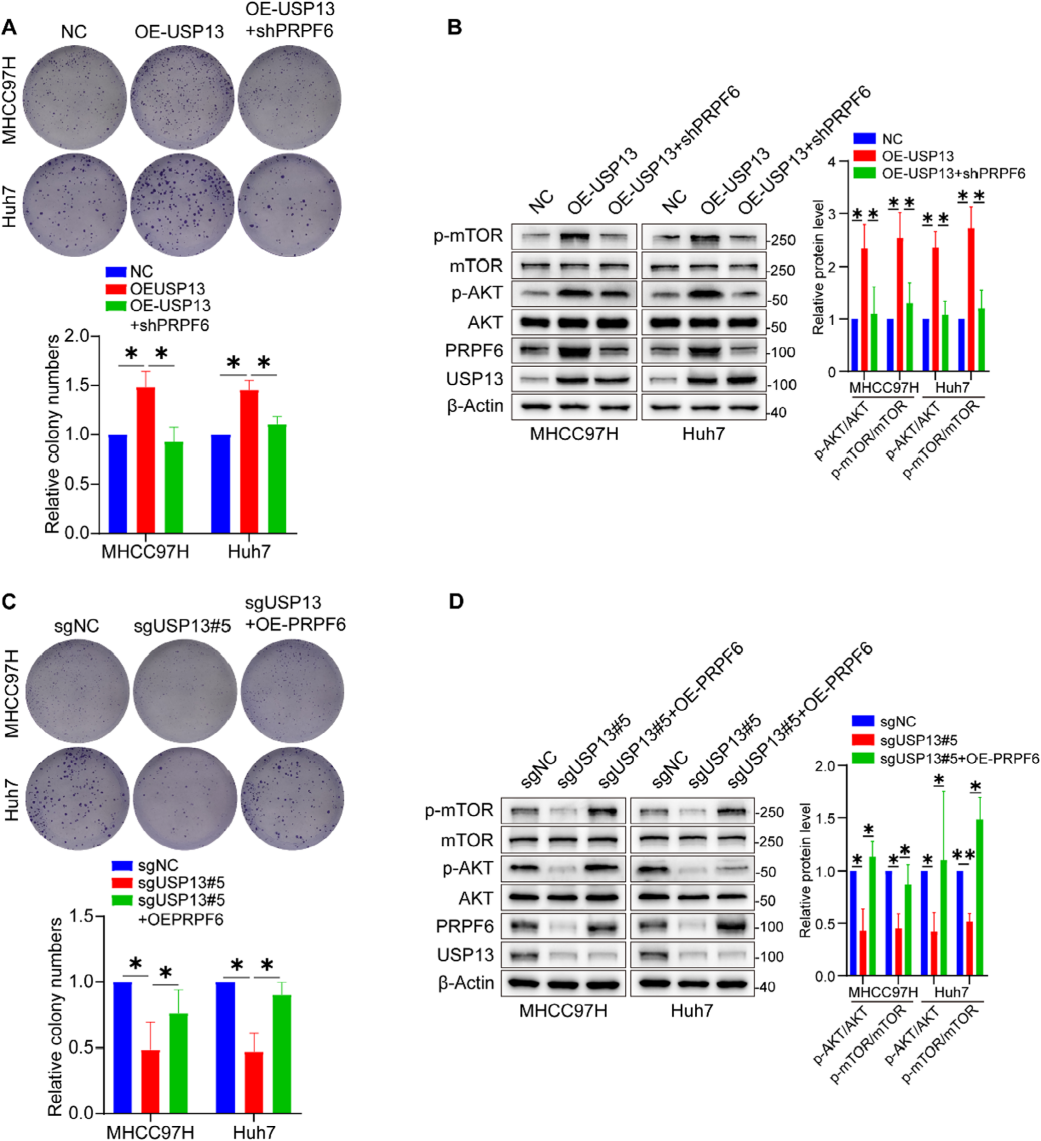
USP13 promotes HCC cell proliferation by regulating PRPF6‐AKT‐mTOR signalling. (A) Colony formation assay showed knockdown of PRPF6 effectively inhibited the cell proliferation induced by overexpression of USP13. (B) Representative blots and quantification showed knockdown of PRPF6 effectively reduced the expressions of p‐AKT and p‐mTOR induced by overexpression of USP13. (C) Colony formation assay showed overexpression of PRPF6 effectively restored the cell proliferation induced by knockout of USP13. (D) Representative blots and quantification showed overexpression of PRPF6 effectively rescued the expressions of p‐AKT and p‐mTOR induced by knockout of USP13. *p < 0.05, **p < 0.01.

To confirm USP13 accelerates the proliferation of HCC cells through regulating PRPF6‐AKT‐mTOR signalling, we performed the rescue experiments by silencing PRPF6 in USP13 up‐regulated cells. It was found that knock down of PRPF6 effectively inhibited the cell proliferation and also reduced the expressions of p‐AKT and p‐mTOR induced by overexpression of USP13 (Figures 7A,B). We further overexpressed PRPF6 in USP13 down‐regulated cells and found that overexpression of PRPF6 effectively restored the cell proliferation and also rescued the expressions of p‐AKT and p‐mTOR induced by knocking out USP13 (Figures 7C,D). These results suggest that the USP13‐PRPF6 axis promotes HCC cell proliferation by regulating AKT‐mTOR signalling.

### 3.6 USP13 Expression Is Correlated With PRPF6 in Human HCC Tissues

**FIGURE 8:**
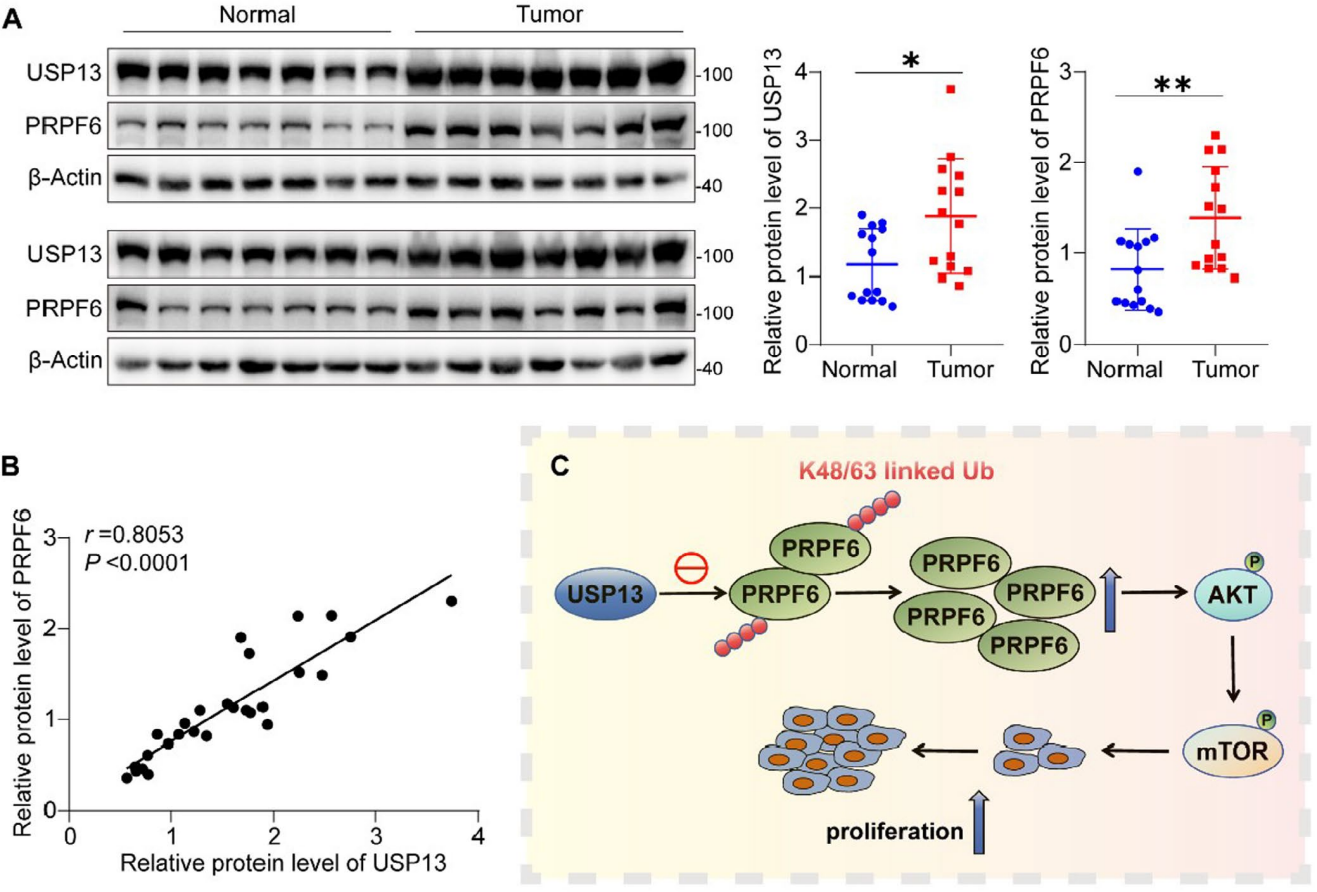
USP13 expression is correlated with PRPF6 in human HCC tissues. (A) Protein levels of USP13 and PRPF6 in 14 normal liver tissues and 14 HCC tissues. (B) Correlation of USP13 and PRPF6 expression in normal liver tissues and HCC tissues. *r* = 0.8053, *p*  ＜ 0.0001. (C) Schematic illustration of this study.

The results above indicate that USP13 deubiquitinates PRPF6, thereby stabilising its protein levels and promoting the proliferation of HCC cells via modulation of the AKT‐mTOR signalling pathway. To further investigate the relationship between USP13 and PRPF6 expression, we conducted a comprehensive analysis of their protein levels in clinical HCC tissue samples. The findings revealed that both USP13 and PRPF6 were markedly overexpressed in HCC tissues and exhibited a robust positive correlation (Figures 8A,B).

## 4 Discussion

As essential regulators in the ubiquitination modification process, DUBs specifically cleave ubiquitin chains to maintain the dynamic equilibrium of the ubiquitin system. This ensures protein homeostasis within cells and supports their normal physiological functions [5, 6]. Research has demonstrated that DUBs play a critical role in the pathogenesis and progression of HCC. For instance, USP1 stabilises CDK5, thereby modulating mitochondrial fission and metabolic reprogramming, which facilitates the progression of hepatocellular carcinoma [8]. Additionally, BRCC36 regulates HMGCR deubiquitination, and inhibiting BRCC36 can suppress liver cancer cell proliferation [9]. Therefore, the identification of differentially expressed DUBs in HCC and the elucidation of their impact on disease onset and progression, along with the underlying mechanisms, may provide valuable insights for the development of targeted therapies in HCC.

The USP family is the largest group of deubiquitinating enzymes known [10]. USP13, a member of this family, regulates key cellular processes such as the cell cycle, DNA damage response and antiviral responses [11, 12, 13]. It has also been linked to the progression of several cancers, including lung squamous cell carcinoma, gastrointestinal stromal tumours and ovarian cancer [14, 15, 16]. In HCC, hypoxia induces USP13 expression, enhancing TLR4 deubiquitination and activating the TLR4/MyD88/NF‐κB pathway, which accelerates tumour growth [18]. However, further research is necessary to fully elucidate the specific role of USP13 in HCC. Here, we have identified that USP13 was significantly overexpressed in HCC and correlated with an unfavourable prognosis. Moreover, our results demonstrated that downregulation of USP13 inhibited HCC cell proliferation, while its overexpression promoted cellular proliferation. These findings suggest that USP13 may serve as a potential biomarker influencing the pathogenesis and progression of HCC.

To further elucidate the molecular mechanism by which USP13 influences the proliferation of HCC cells, we utilised mass spectrometry to identify and characterise the interacting proteins of USP13. Among these, PRPF6 was identified as a potential candidate for further investigation. As an integral component of the U4/U6‐U5 tri‐snRNP complex, PRPF6 is primarily involved in the splicing of precursor mRNA and plays a crucial role in the regulation of gene expression [23]. Mutations in PRPF6 have been implicated in the pathogenesis of retinopathy [24, 25]. In prostate cancer, PRPF6 exhibits elevated expression levels and functions as a key regulator of androgen receptors, thereby enhancing androgen‐induced transactivation to promote prostate cancer progression [26]. In hepatocellular carcinoma, PRPF6 upregulates the androgen receptor signalling pathway, contributing to tumorigenesis [27]. However, the regulatory mechanisms underlying the elevated expression of PRPF6 in liver cancer remain to be fully elucidated. In this study, we demonstrated that USP13 and PRPF6 coexisted within the same protein complex. Our subsequent analyses revealed that USP13 enhanced the stability of PRPF6 by reducing its K48/63‐linked polyubiquitination level, a process mediated by the deubiquitinating activity of USP13. Next, through GSEA, we identified a significant correlation between PRPF6 and the AKT‐mTOR signalling pathway. Further experimental results confirmed that the USP13‐PRPF6 axis promoted the proliferation of HCC cells by modulating the AKT‐mTOR signalling pathway.

In conclusion, we have demonstrated that USP13 can decrease the K48/63‐linked polyubiquitination of PRPF6, thereby stabilising this protein and promoting HCC cell proliferation via regulation of the AKT‐mTOR pathway (Figure 8C). This finding provides a solid foundation for further investigation into the role of USP13 in the pathogenesis and progression of HCC, as well as offering novel insights into the elevated expression levels of PRPF6 protein observed in HCC. However, several key questions remain to be addressed. For example, it is unclear whether differential expression of USP13 affects other malignant behaviours of HCC cells or if there are additional regulatory mechanisms underlying PRPF6 protein overexpression in HCC. These issues warrant further exploration.

## Author Contributions

**Yanyu Jiang:** conceptualization (lead), data curation (equal), formal analysis (equal), supervision (equal). **Qing Luo:** data curation (equal), formal analysis (equal), methodology (lead), software (equal), validation (equal). **Xuanchao Zhang:** conceptualization (equal), data curation (equal), formal analysis (lead), investigation (equal). **Weichao Yang:** methodology (equal), software (equal), visualization (equal). **Renhao Wang:** supervision (equal), validation (equal). **Qinghe Hu:** funding acquisition (equal), supervision (equal), writing – original draft (equal). **Zhiyi Liu:** supervision (equal), validation (equal), visualization (equal), writing – original draft (equal), writing – review and editing (lead). **Bin Zhang:** funding acquisition (lead), project administration (lead), supervision (equal), validation (equal).

## Consent

Written informed consent for publication was obtained from all participants.

## Conflicts of Interest

The authors declare no conflicts of interest.

## Data Availability

All data supporting the findings of this study are available from the corresponding author on reasonable request.
